# Ethno-geographic distribution and histopathological classification of nasopharyngeal carcinoma in a single center in Nepal

**DOI:** 10.1371/journal.pone.0304371

**Published:** 2024-05-31

**Authors:** Subhas Pandit, Simit Sapkota, Abish Adhikari, Prakriti Karki, Deepak Yadav, Roshani Shrestha, Rijendra Yogal, Sanat Chalise, Rakesh Pathak, Anjani Kumar Jha

**Affiliations:** 1 Department of Clinical Oncology, Kathmandu Cancer Center, Tathali, Bhaktapur, Nepal; 2 Department of Radiation Oncology, Kathmandu Cancer Center, Tathali, Bhaktapur, Nepal; 3 Department of Research, Kathmandu Cancer Center, Tathali, Bhaktapur, Nepal; 4 Department of Head and Neck Oncology, Kathmandu Cancer Center, Tathali, Bhaktapur, Nepal; 5 Department of Pathology, Kathmandu Cancer Center, Tathali, Bhaktapur, Nepal; University of Bergen: Universitetet i Bergen, NORWAY

## Abstract

**Introduction:**

Nasopharyngeal carcinoma (NPC) shows geographic and ethnic variation with specific etiopathogenesis. This study characterized the distribution of NPC patients stratified by ethnicity, geography, and histology in a tertiary-level cancer center in Nepal.

**Methods:**

A descriptive retrospective study was designed to analyze NPC cases from different regions among patients visiting the hospital from 2016 to 2021. Demographic and clinical information was obtained from medical records. Ethical approval was granted by the Nepal Health Research Council (NHRC). Data analyses and visualization were carried out with R software.

**Results:**

During the six-year period, a total of 65 individuals were diagnosed with NPC, comprising 42 males and 23 females. Patient median age was 43 years (range 11–85 years). A bimodal age distribution of cases was observed with peaks in patients aged 30–39 years and 50–59 years. Of the NPC patients studied, 29 were from Koshi Province, with 7 cases from Ilam district and 6 cases from Morang district. There were 18 patients in Bagmati Province, and Kathmandu district had the highest number of cases within this region, with 8 patients. The highest proportion of cases were observed among patients of Janajati ethnicity (60%), including Rai, Limbu, and Sherpa people. Histologically, undifferentiated non-keratinizing NPC was the most commonly observed subtype, accounting for 43.1% of cases, followed by 20% differentiated non-keratinizing NPC and 4.6% keratinizing NPC across the entire sample population. The majority of patients (75.3%) were diagnosed at an advanced stage (stage III or IV) with none diagnosed at stage I.

**Conclusions:**

In our study, most cases of NPC occurred in patients from provinces in eastern Nepal (Koshi province), and of the Janajati ethnic community. The most common histological subtype was undifferentiated non-keratinizing carcinoma. Further epidemiological studies could address differences in prevalence and the challenge of late presentation of NPC patients in Nepal.

## Introduction

Nasopharyngeal cancer (NPC) is a distinct type of head and neck malignancy that is known for its unique regional, racial, and biologic characteristics. In 2019, there were 176,501 new cases of NPC worldwide, resulting in 71,610 deaths. NPC has an age-standardized incidence rate of 2.1 and an age-standardized mortality rate of 0.9 per 100,000 persons globally. NPC cases resulted in a total of 2.34 million disability-adjusted life years (DALYs), with an age-standardized rate of 28.0 [1].

Cases of NPC are mainly concentrated in Southern China, South-East Asia, East Asia, the Arctic, and Northern Africa. Such endemicity has been attributed to several etiological factors such as certain environmental and genetic factors, lifestyles, human leukocyte antigen subtypes, and Epstein-Barr virus (EBV) [2, 3]. The clinical presentation, resembling common-cold-like symptoms in early stages, lack of consideration for diagnosis by clinicians, and delay in follow-up have been considered as factors contributing to delayed diagnosis of NPC [4, 5].

The incidence of NPC is two to three times higher in males compared to females [6, 7]. Age-incidence profiles show a bimodal distribution where the risk of NPC is associated with an increase in age up to the first peak in late adolescence or early adulthood, and the second peak in the sixth and seventh decades among low-risk populations, and the absence of an early peak in high-risk populations [8].

Histological studies have shown that the non-keratinizing (NK) type NPC, which has a reasonable prognosis, is more common in countries with a high incidence of NPC. The keratinizing (K) squamous cell type NPC is more common in countries with a low incidence of NPC and has a poorer prognosis [9].

The highest incidence of NPC was noted in Cantonese “boat people” living in South China, intermediate incidence in Southeast Asia (Singapore, Malaysia, Vietnam, Philippines), and rarely occurred in other parts of the world including Korea, Japan, South Asia, and Europe. According to The Global Cancer Observatory (GLOBOCAN) 2020, Asia accounted for 85.2% of the total global incidence of NPC of which approximately 58% is contributed by Eastern Asia and 32.3% by South-Eastern Asia [10].

In the context of Nepal, NPC is ranked 30^th^ in terms of incidence rate and 28^th^ in mortality among different types of cancer [10]. NPC is a major concern in the eastern part of Nepal and accounts for 13.3% of head and neck cancers [11]. Similarly, the neighboring northeastern states of India also reported a high incidence of NPC. The tribes living in this area include migrants from East and Southeast Asia, where substantially higher incidence rates of NPC are reported [12]. The age-standardized NPC rates according to GLOBOCAN 2020 for China, India, and Nepal were found to be 1.0, 0.4 and 0.1 per 100,000 population respectively [10].

Our study at a single tertiary cancer center in Nepal aims to investigate age and gender distribution, geographic location, ethnic background, and histological subtypes of NPC among patients, exploring whether the disease exhibits a distribution pattern comparable to global observations.

## Materials and methods

### Participants

Data from all patients diagnosed with head and neck cancer in the period between 2016 and 2021 were collected from the medical record department of a comprehensive cancer center in the capital of Nepal.

A total of 733 head and neck cancer patients visited the center during the period studied, of whom 65 patients were diagnosed with NPC. Patients were referred from different regions of Nepal except one originating from Darjeeling, India. Nepal is administratively divided into seven provinces, listed from east to west: Koshi (Province 1), Madhesh (Province 2), Bagmati (Province 3), Gandaki (Province 4), Lumbini (Province 5), Karnali (Province 6), and Sudurpaschim (Province 7). These provinces are further subdivided into a total of 77 districts.

Nepal is a multi-ethnic society characterized by its diverse ethnic groups. According to the population monograph of Nepal 2014, the population is divided into six major ethnic groups, specifically Brahmins/Chhetris, Newars, Janajatis, Dalits, Muslims, and Terai/Madhesis [13]. Brahmin/Chhetri is the largest community inhabiting mainly the lower- hills and predominantly speaking Nepali, an Indo-European language. On the other hand, Janajati groups are predominantly Tibeto-Burman peoples residing in the high hills, distinguished by their unique mother tongues and traditional cultures. The Newar people in Nepal exhibit a distinctive social structure, comprising over 40 cultural subgroups divided into Hindu and Buddhist groups, and share a common Newari language. The Dalit community in Nepal refers to a historically marginalized group who faced discrimination and social exclusion. Muslims and Terai/Madhesi people in Nepal predominantly inhabit the southern plains and have distinct religious and cultural identities.

### Data collection and analysis

All patients underwent histological confirmation via biopsy, categorizing their subtypes as keratinizing (K-NPC) or non-keratinizing (NK-NPC) types. NK-NPC was further subcategorized into differentiated-non keratinizing (DNK-NPC) and undifferentiated non-keratinizing (UNK-NPC) subtypes following the 2017 WHO classification [14]. Two expert pathologists were tasked with reviewing pathology slides or reports, and together, they conducted the classification process based on the available materials. The tumor was staged according to the 8th edition of the American Joint Committee on Cancer (AJCC) staging criteria [15]. Most of the patients were treated with different combinations of radiotherapy and chemotherapy.

We established an electronic database containing the demographic information and clinical histories of patients diagnosed with NPC. Data access was limited to authorized personnel, trained on confidentiality. We used secure channels for retrieval and stored data on a password-protected server. All patient data were anonymized to protect privacy and minimizing re-identification risks. Only deidentified data were processed and retained for further research purposes. Rigorous data cleaning procedures were implemented to ensure data quality, involving the removal of duplicated entries and the recovery of missing information using telephonic contact. A data pipeline was developed, which initially used patient addresses to categorize them by district and their respective provinces. Patient surnames were used as an indicator of the distribution of ethnic groups. Subsequently, the data were imported, visually represented through graphs and figures, and underwent comprehensive analysis and interpretation using R software version 4.2.2.

### Ethical considerations

Approval from the Ethical Review Board (Ref no 2125/202) of the Nepal Health Research Council (NHRC) was obtained to carry out this study. The review board accepted a request to exempt the requirement for informed consent, considering the retrospective nature of the study and use of anonymized data.

## Results

### Demographic characteristics

**Fig 1:**
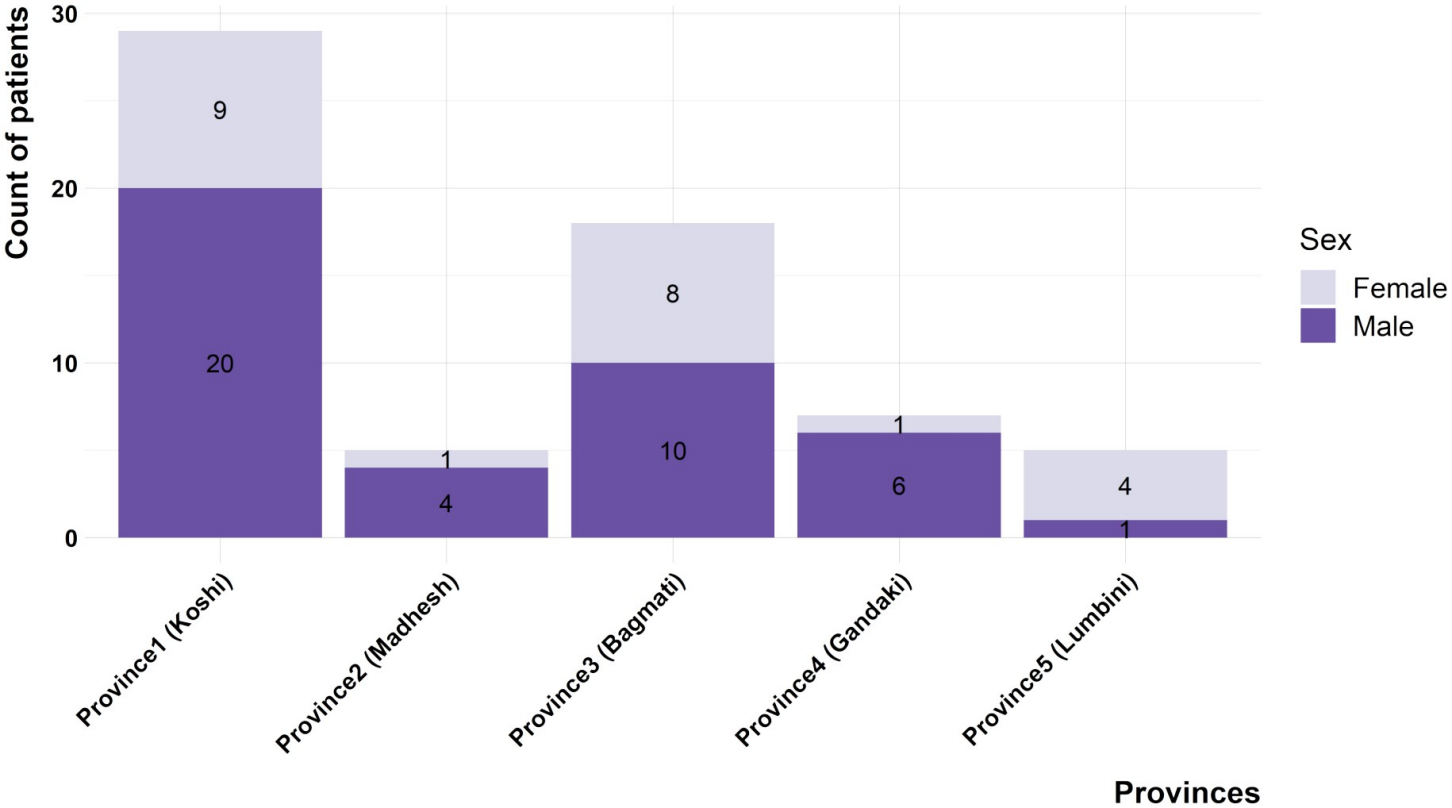
Gender distribution of NPC patients across the provinces of Nepal.

**Fig 2:**
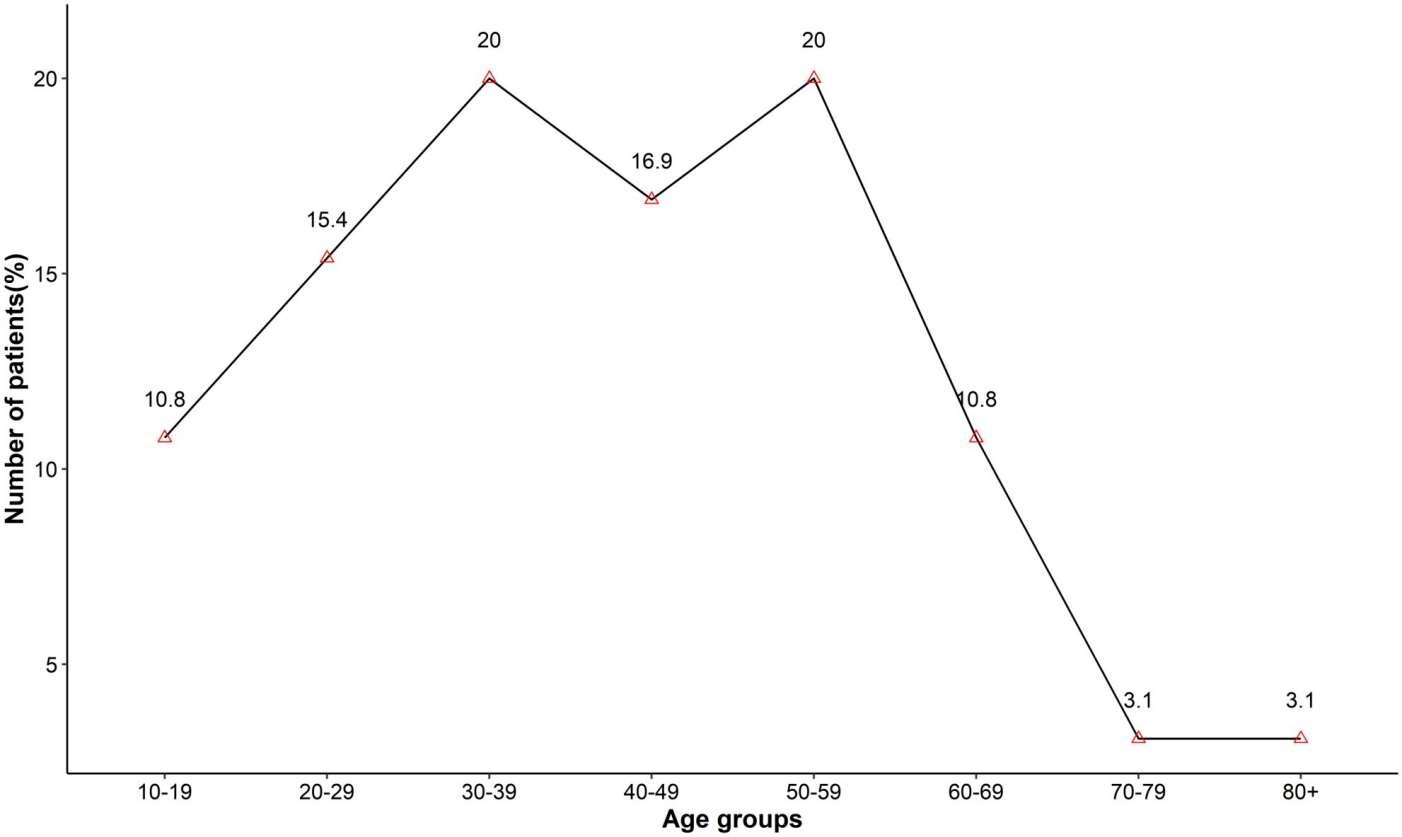
Age distribution of NPC patients.

Overall, 65 patients with NPC were reported in the six-year retrospective study period, constituting 8.8% of a total of 733 head and neck cancer cases. There were higher numbers of males with NPC compared to females, with the former accounting for 64.6% (n = 42) of cases and the latter 35.4% (n = 23), and a male: female ratio of 1.8. Similarly, NPC cases at the provincial level demonstrated a male preponderance except in Lumbini Province (Fig 1). The age of patients ranged between 11–85 years with a median age of 43 years. Bimodal distribution of NPC cases was evident with the earlier peak found to be at the age group 30–39 and the later one at ages 50–59. No cases were reported in patients below 10 years of age in this series (Fig 2).

### Geographic distribution

**Fig 3:**
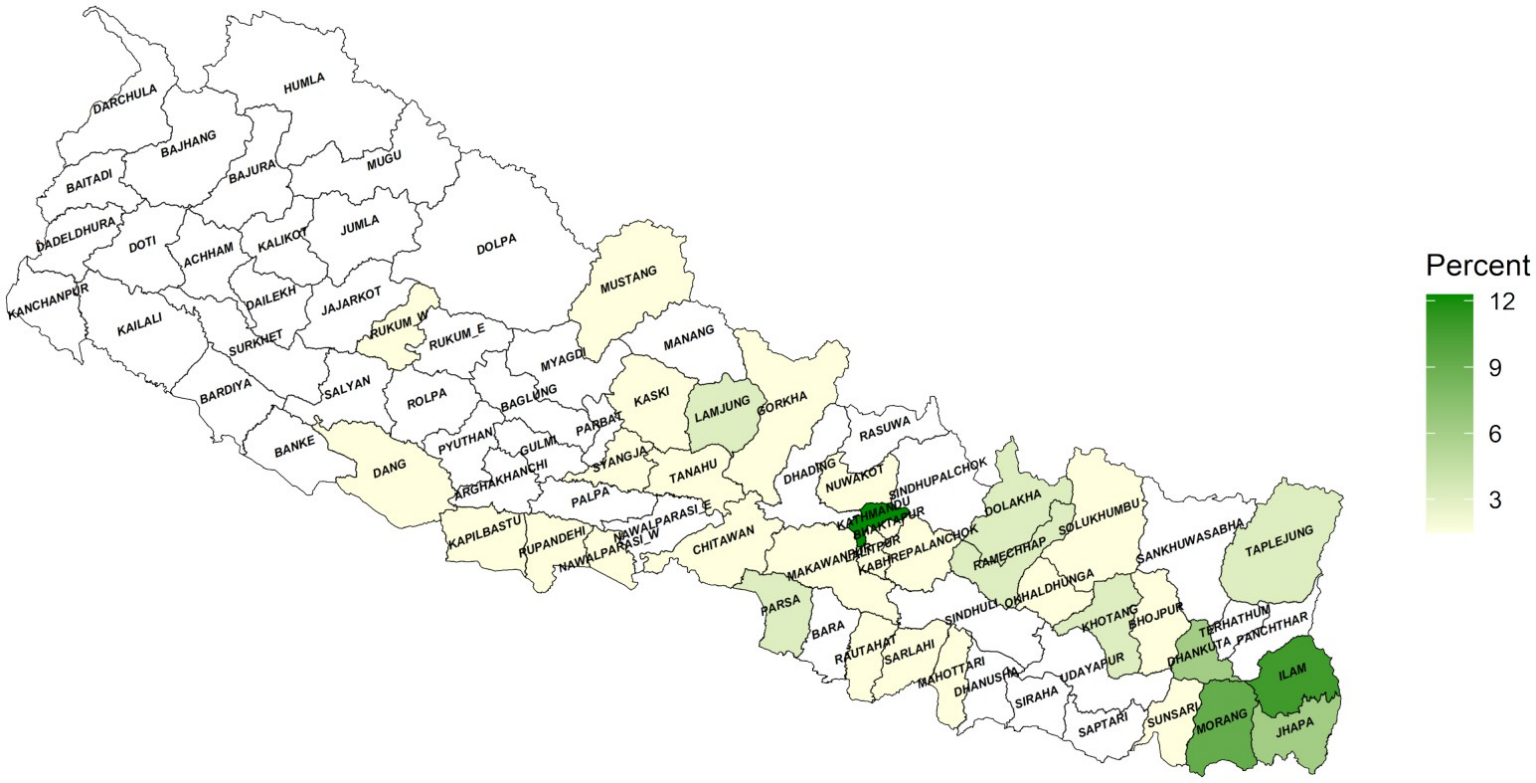
Geographical distribution of NPC in different districts of Nepal. Darker color shades indicate a higher number, lighter shades signify a lower number, and transparent white represents an absence of cases.

In this study series, a concentration of NPC cases was observed in Koshi Province, situated in the eastern region of Nepal. Specifically, 29 patients (representing 44.6% of the total cases) were from Koshi Province, with the highest numbers recorded from the Ilam and Morang districts, recording 7 and 6 cases, respectively. Eighteen patients (constituting 27.7% of the total cases) were from Bagmati Province. The Kathmandu district, Nepal’s capital and a densely populated city, lies in this province and 8 patients (accounting for 44.5% of Bagmati Province cases) were from this district. No patients were recorded from the two western provinces of Nepal, namely Karnali Province and Sudurpaschim Province. (Fig 3).

### Ethnic characteristics

**Fig 4:**
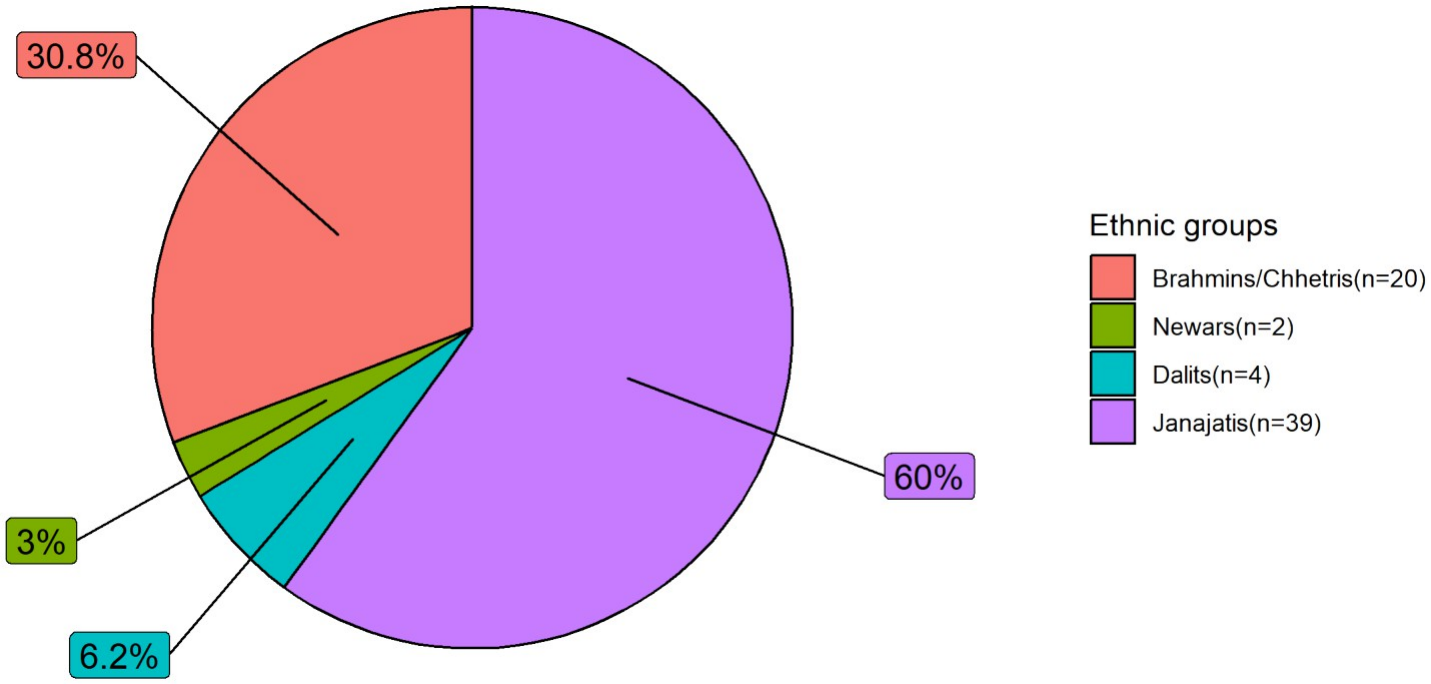
Proportion of NPC cases among different ethnic groups of Nepal.

Janajatis were the most frequenting ethnic group in our series, accounting for 39 (60%) cases followed by Brahmins/Chhhetris (n = 20), Dalits (n = 4), and Newars (n = 2) (Fig 4). There were no cases from the Terai/Madhesis and Muslim categories in our series. Among the Janajatis, specifically, the Rai caste accounted for 23.1% of the total cases (n = 15) followed by Limbus (n = 7), Sherpas (n = 3), Magar (n = 2), and other castes (n = 12).

**Fig 5:**
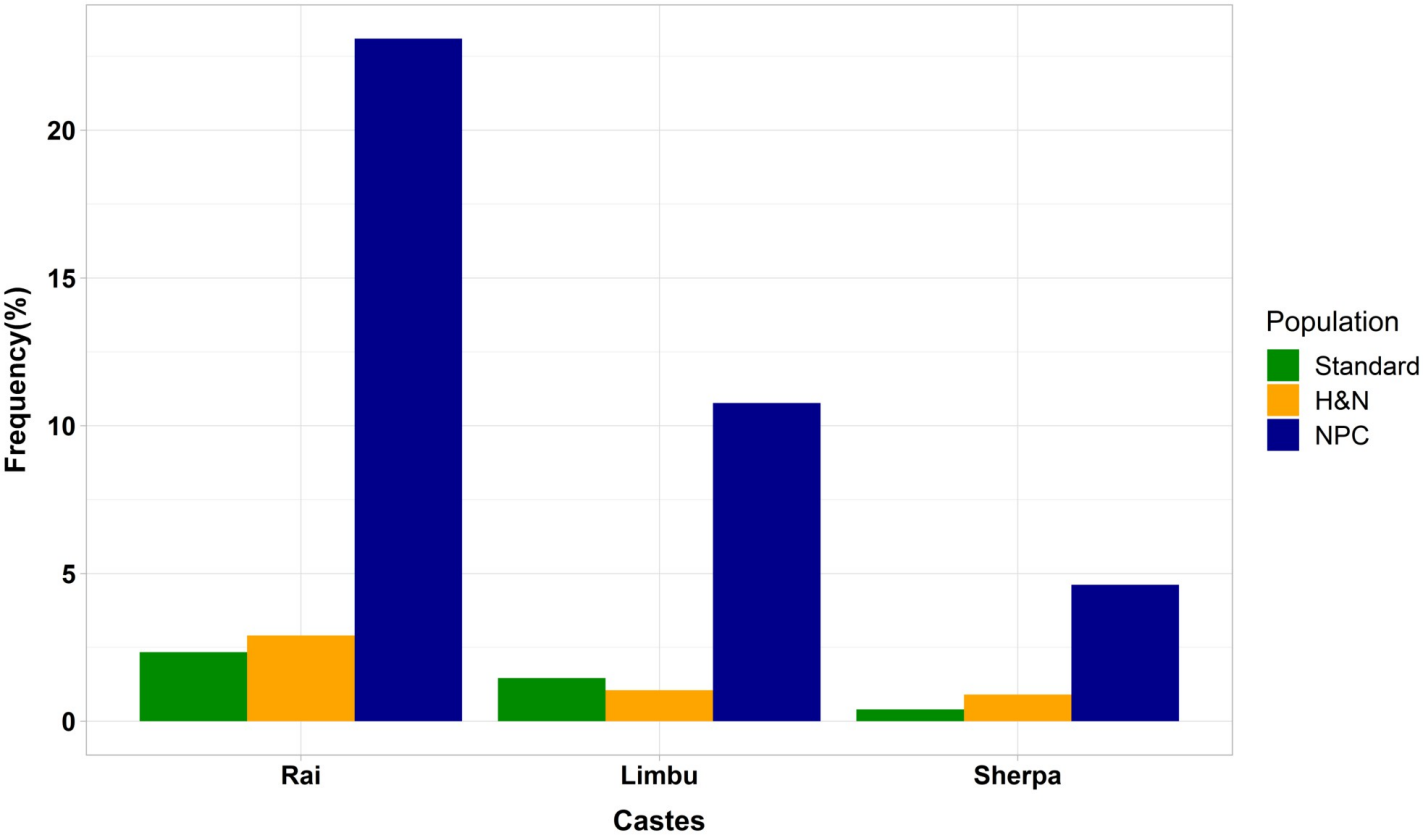
Proportion of different ethnic groups (Rai, Limbu & Sherpa) among the general population, head and neck cancer (excluding NPC), and NPC.

Among the Rai, Limbu, and Sherpa communities, distinct variations in cancer proportions were witnessed. The rate of occurrence of other head and neck cancers (excluding NPC) were similar to those of the of overall population of these communities; however, they demonstrated higher rates of NPC relative to the size of the population than would be anticipated, underscoring the unique nature of NPC in these groups (Fig 5 and Table 1).

**Table 1:** Ethnic disparities in head and neck cancer patterns among Rai, Limbu, and Sherpa communities in Nepal.

Caste	Nepal’s standard population according to Census 2021	Proportion of head and neck cases excluding NPC	Proportion of NPC cases
Rai	6,20,674 (2.2%)	20/668(2.9%)	15/65 (23.1%)
Limbu	4,14,704 (1.4%)	7/668(1.1%)	7/65 (10.8%)
Sherpa	1,30,637 (0.4%)	6/668(0.9%)	3/65(4.6%)

### Staging and histological classification

According to the American Joint Committee on Cancer (AJCC) Staging, most of the patients presented with advanced stages of disease, with many falling into stages III and IV. (Table 2) Only a few were in stage II, and none were diagnosed at stage I.

**Table 2:** Distribution of nasopharyngeal cancer patients by AJCC TNM staging.

TNM AJCC staging	Number	Percentage
Stage I	0	0
Stage II	7	10.7%
Stage III	21	32.3%
Stage IV	28	43.1%
Unknown/recurrence	9	13.8%

The histological subtype was available only for 44 patients. In the remaining patients, the biopsy was performed elsewhere and information about the subtype was not available. The most common subtype was UNK-NPC which was observed in 28 patients (43.1%). DNK-NPC accounted for 13 cases (20%) while K-NPC accounted for 3 cases. UNK-NPC was documented to be the highest number in the Janajati group with a total of 15 cases, followed by Chhetris/Brahmins (10 cases) (Table 3).

**Table 3:** Ethnicity and histological subtypes of NPC patients.

Ethnic Group	Histological Classification				Total
	K-NPC	DNK-NPC	UNK_NPC	Unknown	
**Janajati**	2	7	15	15	39 (60%)
**Brahmins/Chhetris**		5	10	5	20 (30.8%)
**Dalits**		1	3		4 (6.1%)
**Newars**	1			1	2 (3.1%)
**Total**	3 (4.6%)	13(20%)	28 (43.1%)	21 (32.31%)	65

[K-NPC, keratinizing nasopharyngeal carcinoma; DNK-NPC, differentiated non-keratinizing nasopharyngeal carcinoma; UNK-NPC, undifferentiated non-keratinizing nasopharyngeal carcinoma]

## Discussion

Our series showed a specific trend of geographic and ethnic-specific occurrence of NPC. Among the different ethnic groups identified in Nepal, a higher number of NPC patients in our series belonged to Janajatis (60%), followed by Brahmins/Chhetris (approximately 31%), Dalits, and Newars. In the Janajati community, most cases of NPC were found in people from the Rai, Limbu, and Sherpa castes, who live in the mountains and hills of eastern Nepal. Geographical and ethnic disparities can be observed globally in the incidence of the NPC, with a heavy burden primarily confined to Asia, especially among Southern Chinese populations, Southeast Asians, and East Asians, as well as among Chinese immigrants in other regions of the world, while this is rare in other population [2].

This higher number of cases in Janajati population from eastern Nepal might be due to their genetic background, which is influenced by the Himalayan and Adjoining Populations (HAAPs). Studies have shown that HAAPs have unique genetic traits that change gradually as you move across different geographic areas. While HAAPs share characteristics with both East Asian and South Asian populations, they are more genetically similar to East Asian populations. For instance, Sherpas had 78.37±0.65% East Asian ancestry while Subba (Limbu) had 86.47±0.66% East Asian ancestry [16]. Moreover, a study by Basnet et al. found the highest frequency of East Eurasian ancestry in Sherpa (94.3%), while hill Brahmin had around 25% and Hindu Terai accounted for the lowest frequency of East Eurasian ancestry at 8.3% [17]. In our series, the highest number of NPC cases were noted among HAAPs (Rai, Limbu, Sherpa), with intermediate rates among Brahmin/Chhetri, and no cases reported from the Terai population.

The presence of the Himalayas in the northern border of Nepal may act as a formidable barrier to gene flow and human migration. However, the presence of these HAAP populations (also called Tibeto-Burman and Kirantis) in Nepal is likely a result of dispersals that allowed these groups to bypass the Himalayas and reach the region from Tibet directly or via Northeastern India [17]. They are also believed to have migrated from East and Southeast Asia regions known to have a higher prevalence of NPC [18]. For example, Singh et at describe their entry into the eastern hills of Nepal through the Barahakshetra gorge in Koshi Valley [19].

Besides genetics, environmental factors are also known contributors to NPC. The groups with the highest rates of NPC in our cohort come under the classification of Matwali groups, who have a cultural affinity for the consumption of meat and traditional local brews [20–22]. There exists a statistically significant association between total red and processed meat consumption and NPC risk [23]. The production of local brews involves a vigorous fermentation process, which can lead to the formation of nitrosamine, a known dietary carcinogen, during fermentation and storage [24]. Also, cooking practices are likely to alter the concentration of nitrosamine content in the food [25]. Such exposure to carcinogenic compounds may contribute to the higher proportion of cases in Janajatis compared to other ethnic groups. However, Chattopadhyay et. al suggested a strong correlation of NPC risk with genetic factors, rather than with geographical factors [26].

In our study, a male predominance was noted with a male-to-female ratio of 1.83. Intrinsic factors like sex hormones could be attributed to the protective nature of estrogen in females until menopause [27]. A meta-analysis conducted by Long et al. demonstrated a modest association but statistically significant risk of NPC in males but not in females associated with cigarette smoking [28]. Likewise, alcohol consumption is considered a predictor of prognosis in male patients [27]. Both cigarette smoking and alcohol consumption are more widespread among males than females in Nepal. However, several inconsistent findings have been linked with alcohol intake as a risk factor for NPC that may be due to differences in study parameters and consideration of confounding factors [29]. Hence, besides genetic basis, exposure to extrinsic factors is likely to pose a greater risk of NPC in males.

A diverse distribution of NPC from pediatric to geriatric age groups was observed in our study. In our series, no cases were recorded in children younger than 10 years of age as NPC being a rare malignancy in the pediatric age group. Our study exhibits a bimodal age pattern, which is consistent with a low-incidence population, however, there is a contrast in the specific age groups where the peaks occur. In our study, the first peak was observed in patients in their fourth decade, and a second peak emerged in the sixth decade. In contrast, low-incidence populations typically have their first peak in early adulthood (15–24 years old), followed by a second peak in patients aged 65–79 years [8].

Our findings on the WHO subtype align with previous research in endemic regions of NPC, such as Hong Kong and Northern Morocco, where over 95% of NPC cases were classified as NK-NPC whereas K-NPC is more common in sporadic areas [7, 30]. In our study, we observed that Janajatis, a group with a higher proportion of cases, predominantly presented with UNK-NPC, consistent with the common subtype in endemic regions [31, 32]. The closest association is seen between EBV infection and UNK-NPC [33], while tobacco smoking and alcohol consumption also contribute to NK-NPC [34]. The majority of the cases presented at the advanced stage i.e., Stage IV followed by Stage III. Presentation of patients with advanced stage is often correlated with silent symptoms and insidious site [3].

This study has a number of limitations. It is important to recognize that this is a single-center study with a relatively small sample size, which may restrict the generalizability of our findings to the broader population of the entire country of Nepal. There is also a lack of information about the individual patients’ lifestyles. The EBV titer, which correlates with causation, prognosis, and treatment monitoring for NPC, was not available for analysis. Additionally, we observed instances where specific pathology subtypes were missing from biopsy reports, underscoring the importance of adhering to standardized pathology reporting protocols in the future. Despite its limitation, this is the first study from Nepal investigating ethnic and geographical distribution which contributes valuable insights into the understanding of NPC patterns in Nepal. Future research could explore the nuanced interplay of genetic, environmental, and lifestyle factors and help in guiding targeted prevention and intervention strategies to alleviate the burden of NPC in the region.

## Conclusions

Our series notes a greater occurrence of NPC in patients from the Eastern provinces of Nepal, particularly among the Janajati ethnic group, including subgroups like Rai, Limbu, and Sherpa population. UNK-NPC was the main histological subtype in our series. Further studies involving multiple centers and population-based cancer registries can confirm our findings regarding the role of environmental and genetic factors in NPC development.

## Supporting information

S1 Checklist STROBE checklist v4 combined Plos medicine. (DOCX)
